# Identifying key factors contributing to treatment costs for snakebite envenoming in private tertiary healthcare settings in Tamil Nadu, India

**DOI:** 10.1371/journal.pntd.0011699

**Published:** 2023-10-16

**Authors:** Anika Salim, Jarred Williams, Samir Abdel Wahab, Tade Adeshokan, José R. Almeida, Harry F. Williams, Rajendran Vaiyapuri, Subramanian Senthilkumaran, Ponniah Thirumalaikolundusubramanian, Ketan Patel, M. Fazil Baksh, Matthew R. Lewin, Sakthivel Vaiyapuri

**Affiliations:** 1 School of Pharmacy, University of Reading, Reading, United Kingdom; 2 Toxiven Biotech Private Limited, Coimbatore, Tamil Nadu, India; 3 Manian Medical Centre, Erode, Tamil Nadu, India; 4 The Tamil Nadu Dr M.G.R Medical University, Chennai, Tamil Nadu, India; 5 School of Biological Sciences, University of Reading, Reading, United Kingdom; 6 Department of Mathematics and Statistics, University of Reading, Reading, United Kingdom; 7 California Academy of Sciences, San Francisco, California, United States of America; Fundação de Medicina Tropical Doutor Heitor Vieira Dourado, BRAZIL

## Abstract

**Background:**

India suffers ~58,000 annual deaths due to snakebites. The ‘Big Four’ snakes (Russell’s viper, Indian cobra, common krait, and saw-scaled viper) that are responsible for most bites cause diverse clinical effects. Delayed treatment increases the risk of serious complications and treatment costs. Although government hospitals offer free treatment for snakebites in India, most patients opt for private healthcare, which is an out-of-pocket expense as they often lack health insurance coverage. This study aims to analyse snakebite treatment costs in private tertiary care hospitals in Tamil Nadu, India and identifies the key factors contributing to treatment costs.

**Methodology/Principal findings:**

The treatment cost details for 913 snakebite victims were collected from 10 private tertiary care hospitals across Tamil Nadu. The data were classified into hospital, pharmacy, investigation, and laboratory costs, and analysed to determine various factors that contribute to the costs. The results demonstrate that the average treatment costs vary widely for different snakes. The hospital and pharmacy costs are higher than investigation and laboratory costs for all snakebites. Notably, Russell’s viper bites cost significantly more than the bites from other snakes. Overall, the type of snake, nature of complications, specialist treatments required, and arrival time to hospitals were identified as some of the key factors for higher treatment costs.

**Conclusions/Significance:**

These data demonstrate that ~80% of snakebite patients can be treated with INR 100,000 (~GBP 1000 or USD 1200) or less. This study emphasises the urgent need to improve rural medical care by providing appropriate training for healthcare professionals and essential resources to facilitate early assessment of patients, administer the initial dose of antivenom and refer the patients to tertiary care only when needed. Moreover, the outcome of this study forms a basis for developing appropriate policies to regulate snakebite treatment costs and provide affordable medical insurance for vulnerable communities.

## Author summary

Snakebite envenoming (SBE) predominantly affects poor communities living in rural areas of developing countries. As SBE induces a wide range of pathological effects in patients, they need a broad spectrum of treatment approaches to tackle those issues. Therefore, antivenom alone is not sufficient to treat SBE patients. In India, most people (including SBE patients) seek treatments from private healthcare settings, although government hospitals provide free treatments. Hence the treatment costs pose a significant burden on the SBE victims and their families leading to severe socioeconomic impacts. To develop better policies to support the clinical management of SBE, it is critical to estimate the costs of SBE treatment in private healthcare settings. In this study, we analysed the treatment costs of 913 snakebite patients who were treated in 10 different private tertiary care hospitals in Tamil Nadu, India, and identified various factors that contribute to treatment costs. For example, snake type, specialist treatments and the time of arrival to the hospital following bites are some of the key factors leading to increased treatment costs. These results form the basis for developing policies to regulate SBE treatment costs, provide health insurance coverage and ensure timely treatment for SBE victims in rural healthcare settings.

## Introduction

Snakebite envenoming (SBE), a high-priority neglected tropical disease [1], is considered a disease of poverty as it predominantly affects rural populations living in developing countries such as India [2–5]. SBE has been estimated to cause around 150,000 deaths and 500,000 permanent disabilities every year worldwide [2, 6, 7]. India alone accounts for around 58,000 deaths every year due to SBE [8,9]. A household survey in Tamil Nadu, India [10] found that up to 79% of envenomings occurred when patients were performing agricultural fieldwork and SBE induces significant socioeconomic impacts. However, the true magnitude of SBE and its socioeconomic ramifications on rural communities are not yet fully understood [2,10,11]. In India, the ‘Big Four’ snakes [Russell’s viper (*Daboia russelii*), Indian cobra (hereafter, referred to as cobra) (*Naja naja*), common krait (*Bungarus caeruleus*), and saw-scaled viper (*Echis carinatus*)] are responsible for most of the incidents, resulting deaths, and disabilities [10,12–14]. Notably, many patients do not seek appropriate hospital treatment promptly after bites as they rely on locally available traditional healers and a range of often dangerous/inappropriate first aid [10,12,15,16]. The challenges in accessing transport facilities, emergency services and correct treatments in rural areas are also major constraints in seeking prompt treatment for SBE [10,16–18]. These factors contribute to significant delay in seeking treatment and result in worse-than-necessary complications which pose additional challenges for the clinical management of SBE as well as subsequent economic losses for generations [10,14,16,18].

Although antivenom is essential for treating SBE patients, it is not the only treatment that can save lives [18–20]. For example, airway and respiratory compromise in SBE patients suffering from neurotoxic envenomings can be managed almost entirely by mechanical ventilation where no antivenom is available or the antivenom is not effective [20–23]. Tissue necrosis around the bite site may require operative interventions such as local debridement and fasciotomy along with possible amputation where attempts at limb salvage are not viable or successful [18,19]. A wide range of antibiotics is used to combat bacterial infections arising from SBE and patients who experience acute kidney injury often need dialysis [20,24–26]. Thus, a notable variety of treatment methods is required to counteract the broad spectrum of clinical effects induced by SBE. All these treatment methods increase the final treatment costs for SBE patients. In countries like India, a significant proportion of people prefer to seek medical treatment in private healthcare settings even though the state and/or central governments provide free treatment in their healthcare centres [10,12,15,18,27]. Most rural people in India do not have health insurance policies to cover their medical treatment [10,12].

Since SBE largely affects agricultural populations who struggle to earn enough money for their everyday survival, the treatment costs for a single SBE event can significantly alter their lives through resulting socioeconomic impacts [4,10]. Some of the socioeconomic impacts include the loss of or changing occupation, inability to work, loss of properties, jewellery, and savings, and removing children from their education to send them to work to meet the family’s financial needs [10,28]. Notably, some people are reluctant to seek hospital treatments only because of the high treatment costs, and certain people regret taking hospital treatments after surviving SBE because of the long-term financial consequences [10]. Hence, it is critical to not only save their lives from SBE but also minimise the treatment costs and resulting socioeconomic ramifications. SBE treatments are mostly provided by tertiary care hospitals that possess the necessary facilities, equipment, and expertise [18]. Hence, the treatment costs may vary based on the settings that the SBE patients choose, and often they visit more than one facility [18]. However, to improve clinical management and develop relevant policies, it is critical to understand the various factors involved in the treatment costs for SBE in private healthcare settings. To the best of our knowledge, no specific studies were performed to address this gap in the existing knowledge to determine the average treatment costs for SBE, and their justification, and identify the key factors contributing to high treatment costs.

To address this critical issue in the SBE field, this study aimed to determine the direct costs of treating SBE patients and key factors that increase treatment costs by analysing data from a large (913) cohort of SBE patients who were treated in different private tertiary care hospitals across Tamil Nadu, India. The data from this study indicates that on average, most SBE patients can be treated with roughly INR 100,000 (~GBP 1000 or USD 1200) or less, and hospital and pharmacy costs play critical roles in overall treatment costs. These results shed light on this poorly studied aspect of SBE and create awareness amongst SBE patients, clinicians, healthcare policymakers and insurance providers about the significance of timely treatments for SBE, the various components involved in clinical management and the justification for treatment costs.

## Methods

### Ethics statement

This study was approved by the Institutional Ethical Review Committees at Toxiven Biotech Private Limited, Tamil Nadu (reference number: 2019-001/002) and the University of Reading (reference: UREC 23/05). The study was performed in line with the guidelines provided by the Indian Council for Medical Research and the Declaration of Helsinki. Before enrolment in this study, informed written consent was obtained from every participant or their legal guardians.

### Patient and public involvement statement

The patients and members of the public were not directly involved in the study design, data collection, analysis and writing of the manuscript mainly due to their limited availability to attend several meetings that are often arranged during day times when they are also occupied with their daily work. However, all patients provided written consent to collect these data and to publish them in scientific journals. Involvement in this study was completely voluntary and participants could withdraw their consent at any time during this study. The participation did not result in any implications for their treatment and outcomes. We will ensure that the results of this study are disseminated to study participants and wider communities through scientific publications, which might be followed by press releases in media in the local language and English.

### Study design and data collection

This study was carried out in 10 different private tertiary care hospitals located in various cities/towns across Tamil Nadu (a large state in South India with a high burden of SBE) in India. These hospitals were not selected to represent the whole state although they are distributed across the state. They were selected using the convenience sampling approach mainly due to their popularity in treating snakebites and easy access to snakebite patients. Indeed, four of them are snakebite (Note: the term ‘SBE’ refers to actual envenoming and ‘snakebite’ includes all bites including dry and non-venomous bites) referral hospitals where patients are routinely referred from other healthcare settings. The data were equally distributed across four snakebite referral hospitals (150 from each) and other hospitals (50 from each except one hospital where 63 patients were recruited due to the availability of many patients bitten by snakes other than Russell’s viper). The hospital authorities wish to remain anonymous due to the sensitive nature of the data provided on treatment costs, and therefore, we are unable to reveal their names, precise locations, and treatment costs in individual hospitals. Data were collected between January 2019 and June 2021. All the data sets were received in an anonymised format without any personal details of the patients.

All patients bitten by venomous species presenting at the hospital were assigned a patient number, so their details could be anonymised. In total, 927 patients were admitted following venomous bites, of which 913 suffered from snakebites, 1 centipede, 9 unknown insect bites and 4 scorpion bites. Patients who were bitten by non-snake species were excluded from this study. Hence, a total of 913 patients were included for further analysis. The details of their treatments (except basic details to ascertain if they visited another medical facility and received any treatment specifically antivenom) and costs before admission to these hospitals were not collected in detail as this was not the scope of this study. Therefore, this study presents only the data that were collected from the study hospitals.

All the patients included in this study were subjected to detailed clinical examination and laboratory investigation at the time of arrival at the emergency department of each hospital. Demographic data and patient anamnesis upon presentation were collected. In some cases, the patients or their relatives brought dead or live snakes for identification. A range of clinical symptoms (a total of 45) was determined by clinicians to be used for clinical assessments. Patients were treated according to each hospital’s standard of care including antivenom administration and supportive measures.

### Classification of patients

The offending snake species was determined mainly based on the presenting clinical symptoms and the dead/live specimens brought to the hospitals in some cases and categorised as Russell’s viper, saw-scaled viper, common krait, cobra, or non-venomous snakes. Since the clinicians in selected hospitals treated snakebite victims for several years, they were familiar with the common symptoms of envenomation from Big Four snakes. However, in cases where clinicians could not clearly identify the snake species based on the information provided by patients or their relatives and presenting symptoms, the bite was classified as an unknown category. In some cases, the patients were shown images of commonly available snakes around their regions and asked to identify the offending species. While this approach helped to identify the offending snake in some cases in line with their symptoms, in other cases, this was not helpful. For example, patients who identified multiple snakes as potentially responsible for the bite were categorised as being of unknown origin. Therefore, this classification method aided in identifying appropriate offending species to calculate their treatment costs as accurately as possible.

### Classification of clinical manifestations

Upon consultation with clinicians, a range of clinical signs and symptoms were reviewed to provide the appropriate level of diversity in symptomology following bites from Indian venomous snakes. These include the presence of puncture wounds/fang marks, bleeding, pain, changes in blood pressure, muscle weakness, blurred vision, vomiting, numbness, swelling, sweating, ptosis, skin discolouration, difficulty breathing, necrosis, epigastric burning sensation, giddiness, difficulties in swallowing, absence of urine output, cellulitis, generalised itching, haematuria, abdominal pain, fever, chills, unhealing wound, burning sensation at the bite site, sore throat, heaviness of head, chest pain, tickling sensation, tiredness, restlessness, haematemesis, haemoptysis, dyspnoea, diplopia, oliguria, tremors, shivering, xerostomia, frothing, cold extremities, pulmonary oedema and gum bleeding. The total number of signs and symptoms experienced by each victim was analysed in this study.

### Classification of total treatment costs

The direct treatment cost data were divided into four categories. (1) Hospital cost which was defined by the patient’s duration of hospitalisation and the type of procedures/treatment received (including intensive care unit, ward stay, use of medical equipment e.g., ventilation support and operative interventions). (2) Pharmacy costs which covered the cost of medications received including antivenom, antibiotics and blood products along with consumables used during their treatment. (3) Investigation costs were the fees charged for the care provided by the healthcare team including the expenses charged by specialist clinicians to visit the patient from other organisations/hospitals. (4) The laboratory costs covered the expenses for different laboratory tests (such as haematological analysis and microbial culture) and specialist investigations [e.g., computed tomography (CT), magnetic resonance (MRI) and ultrasound imaging] conducted on the patients. A total treatment cost was calculated by adding up each of these individual parts. All the prices charged to participants in this study were mentioned in the form of Indian rupees (INR).

### Statistical analysis

A generalised linear model with a gamma distribution and a log-link function for the mean component was used to model treatment costs with the snake, age, sex, number of vials of antivenom and interactions as covariates. This modelling approach is due to the fact that the treatment costs are positive, and it allows us to address the observed heterogeneity in the data detected during the model validation process. Before fitting any model, data exploration was applied using the protocols that were previously described [29, 30]. The presence of outliers and collinearity were investigated. One way ANOVA was performed to compare the treatment costs for different snake species. The Wald test was used to evaluate the effects of gender, age, and the number of antivenom vials on total treatment costs. The descriptive analysis was used for calculating the mean, median and standard deviation for different variables. Similarly, percentages, ranges and ratios were calculated to summarise the distribution of data where necessary.

All statistical analyses were performed using the R (Version 4.1.2, R Foundation for Statistical Computing, Vienna, Austria) and SPSS (Version 27, IBM, USA) statistical packages and GraphPad Prism (Version 7, GraphPad Software, San Diego, CA, USA).

## Results

### Characteristics of the study population

**Fig 1:**
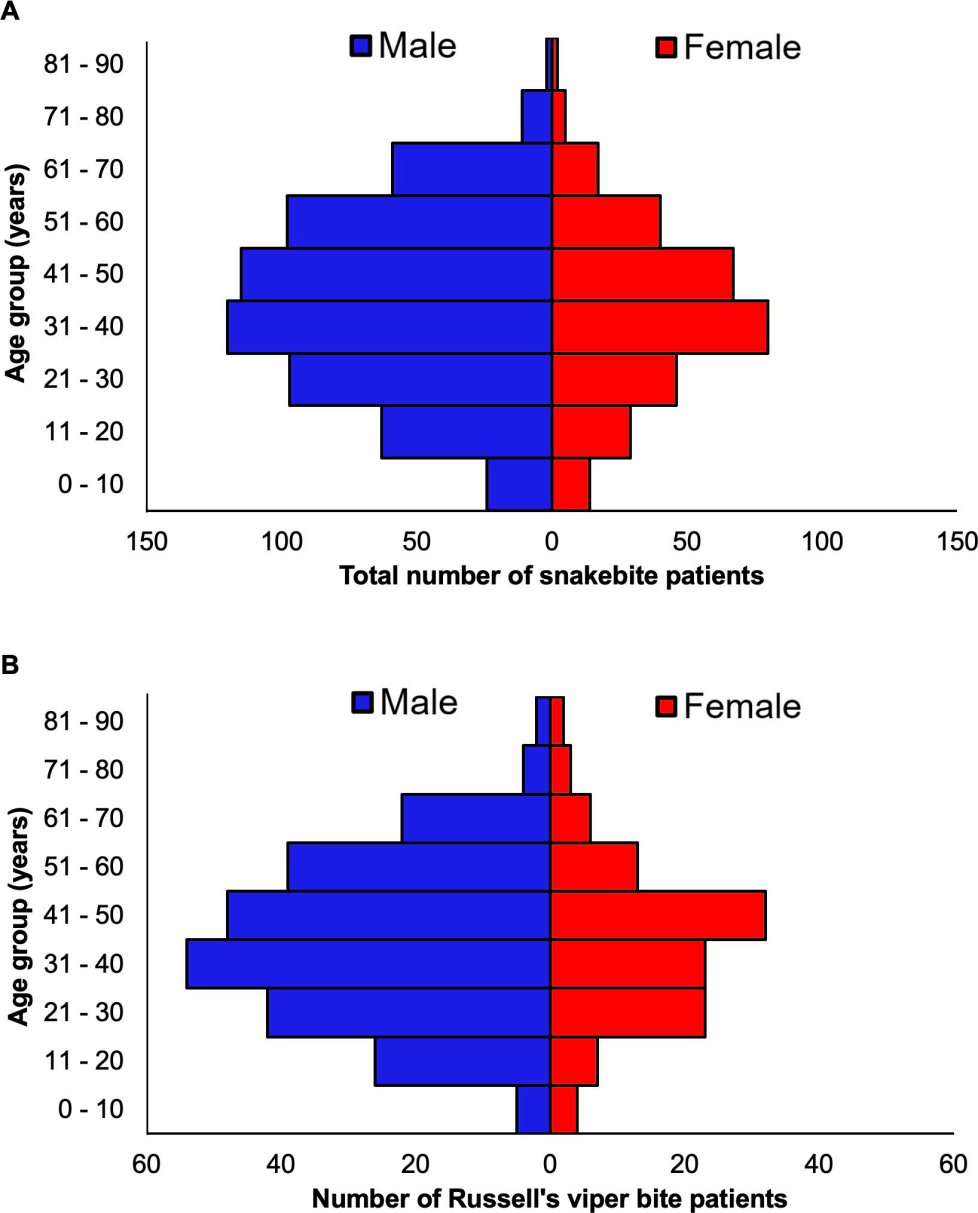
Age and gender profile of snakebite patients. (**A**) A graph showing the age and gender distribution of the 913 total snakebite patients included in this study. (**B**) The age and gender distribution of 355 Russell’s viper bite patients.

In total, 913 snakebite patients who were admitted to 10 different private tertiary care hospitals in Tamil Nadu were included in this study (**Fig 1A**). Among the study population, 600 (65.7%) males and 313 (34.3%) females were included indicating that males are more vulnerable to snakebites than females, probably due to their increased involvement in agricultural activities. Further classification of these data based on the type of snake involved in the incident revealed that 355 [38.9% of total incidents: 242 (68.2%) males and 113 (31.8%) females] patients were bitten by Russell’s vipers (**Fig 1B**), 133 [14.6% of total incidents; 103 (77.4%) males and 30 (22.6%) females] patients were bitten by common kraits, 108 [11.8% of total incidents; 70 (64.8%) males and 38 (35.2%) females] patients were bitten by saw-scaled vipers and 69 [7.6% of total cases; 43 (62.3%) males and 26 (37.7%) females] patients were bitten by cobras. Moreover, 87 [9.5% of total incidents; 47 (54%) males and 40 (46%) females] patients were bitten by non-venomous snakes such as Indian rat snakes (*Ptyas mucosa*), wolf snake (*Lycodon aulicus*), and common bronzeback tree snake (*Dendrelaphis tristis*). A total of 161 patients [17.6% of total cases; 95 (59%) males and 66 (41%) females] were bitten by unidentified snake species as their identities could not be ascertained based on the available details. This category may include a few cases of Malabar (*Craspedocephalus malabaricus*), hump nosed (*Hypnale hypnale*) and bamboo (*Craspedocephalus gramineus*) pit vipers as these cases are also being reported in distinct parts of Tamil Nadu (information gathered from collaborating hospitals).

To determine the specific age groups that are most affected by snakebites, the data were categorised based on the age of the patients. In total, 38 (4.2% of total incidents) people were in the age range of 0–10, 92 (10%) in 11–20, 143 (15.7%) in 21–30, 200 (21.9%) in 31–40, 196 (21.5%) in 41–50, 146 (16%) in 51–60, and 98 (10.7%) were above 60 years old (**Fig 1A**). The youngest patient included in this study was 2 years old and the oldest patient was 91 years old with a median of 40 years (IQR: 28 to 51; SD = 16.67) and a mean of 39.9. For Russell’s viper bites, the minimum age was 2.5 years old, and the maximum age was 91 with a median age of 40 and a mean of 40.6 (IQR 29 to 51; SD = 16.2) (**Fig 1B**). For common krait bites, the minimum age was 11 and the maximum age was 81 with a median age of 47 and a mean of 46.9 (IQR 38 to 55; SD = 13.9). For cobra bites, the minimum age was 4 years old, and the maximum age was 80 with a median age of 35 and a mean of 36.2 (IQR 21 to 52; SD = 18.5). For saw-scaled viper bites, the minimum age was 2 years old, and the maximum was 70 with a median age of 41.5 and mean of 41.8 (IQR 31 to 54; SD = 15.4). For non-venomous snakebites, the minimum age was 3 years old, and the maximum age was 75 with a median age of 33 and a mean of 32.2 (IQR 19.5 to 31.9; SD = 16). For unknown snakes, the minimum age was 2 years old, and the maximum age was 75 with a median age of 39 and a mean of 37.2 (IQR 24 to 50; SD = 17.7). This data indicates that males of working age (from 21 to 60) are most likely to suffer snakebites.

We then analysed the most common signs and symptoms that the patients displayed upon admission to the study hospitals. Russell’s viper patients experienced pain [342 (96.3% of total Russell’s viper bites)], swelling [286 (80.6%)], vomiting [173 (48.7%)], blurred vision [119 (33.5%)] and bleeding [117 (33%)] as the most prevalent signs/symptoms. Common krait bites induced ptosis [62 (46.6% of total krait bites)] and blurred vision [50 (37.6%)]. Cobra bites induced pain [67 (97.1% of total cobra bite patients)], swelling [42 (60.9%)], vomiting [28 (40.6%)], blurred vision [22 (31.9%)] and ptosis [21 (30.4%)]. Saw-scaled viper bites induced pain [107 (99% of total saw-scaled viper patients)], swelling [66 (61.1%)], and blurred vision [30 (27.8%)]. Non-venomous snakes mainly induced pain [72 (82.8% of total non-venomous cases)]. Bites from snakes within the unknown category induced pain [131 (81.4% of unknown bite cases)], swelling [84 (52.2%)], vomiting [54 (33.5%)], and bleeding [30 (18.6%)].

### Russell’s viper bites incur higher treatment costs than bites from other snake species

**Fig 2:**
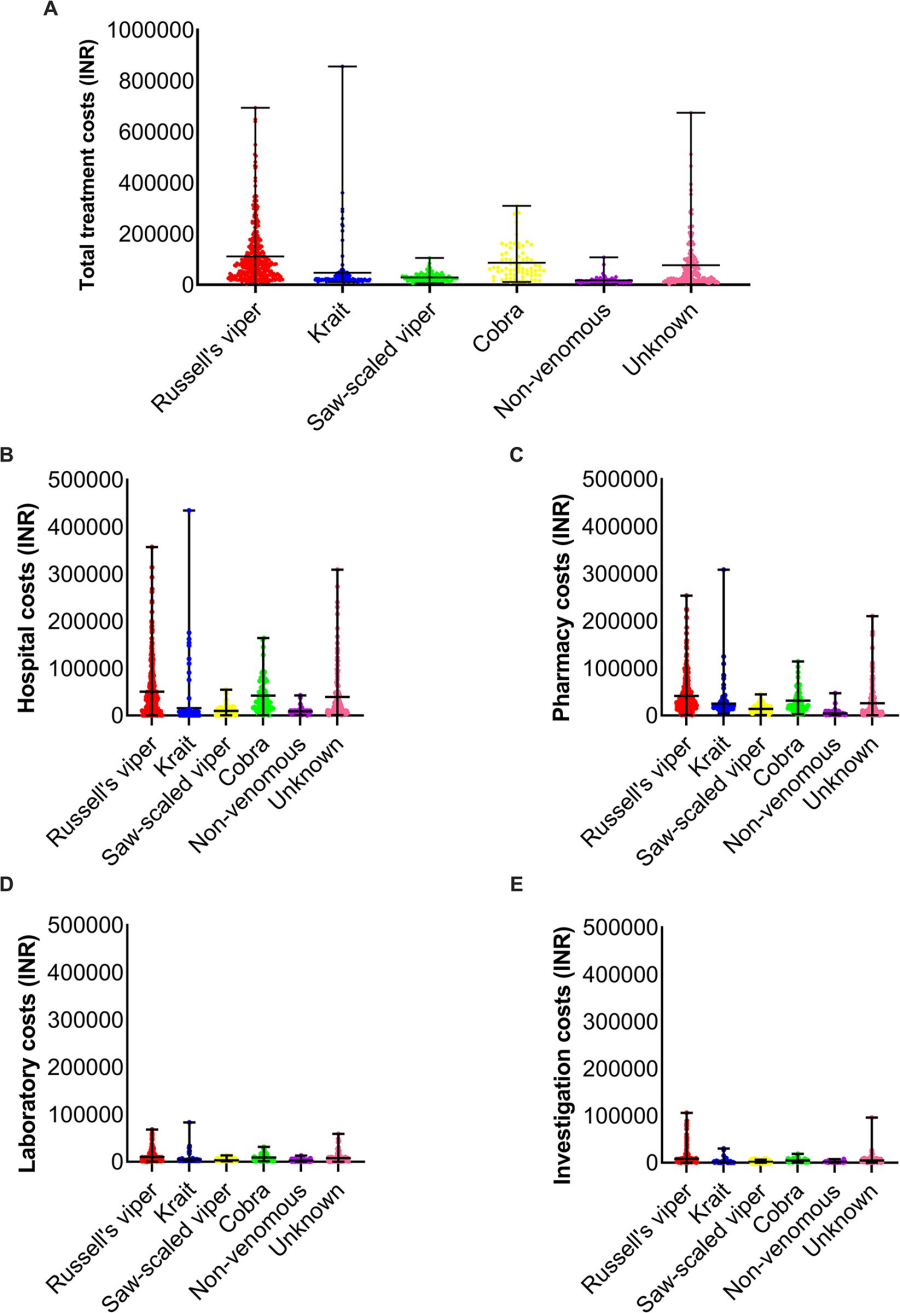
The breakdown of total treatment costs based on the type of snake species and nature of expenses. (**A**) Total treatment costs of patients bitten by different snake species. The total costs for the hospital (**B**), pharmacy (**C**), laboratory (**D**) and investigation (**E**) were also analysed individually.

The total treatment costs were classified into hospital, investigation, laboratory, and pharmacy costs. The total treatment costs for Russell’s viper bites ranged from INR 2,502 to INR 694,620 with an average of INR 111,496 (median value of INR 79,260) (**Fig 2A**). The costs for common krait bites ranged from INR 10,504 to INR 856,688 with an average cost of INR 47,276 (median of INR 21,378). For cobra bites, the costs ranged from INR 11,029 to INR 309,683 with an average of INR 86,833 (median of INR 63,893). Treating saw-scaled viper bites costs between INR 5,814 to INR 105,223 with an average of INR 28,863 (median of INR 24,546). The costs of non-venomous bites ranged from INR 2,937 to INR 108,057 with an average of INR 17,554 (median of INR 15,162). The unknown snake category costs ranged from INR 2,101 to INR 674,884 with an average of INR 77,342 (median of INR 36,804). These data suggest that the average treatment costs for Russell’s viper bites are higher than the costs for treating bites from other snake species.

Further evaluation of treatment costs revealed that hospital (**Fig 2B**) and pharmacy (**Fig 2C**) costs are considerably higher than the laboratory (**Fig 2D**) and investigation (**Fig 2E**) costs. For example, the hospital costs ranged from INR 500 to INR 357,300 (average of INR 50,739) and pharmacy costs ranged from INR 1,111 to INR 253,713 (average of INR 41,638) for Russell’s viper patients. However, the average costs of investigation (INR 100 to 106,000) and laboratory tests (INR 191 to 68,307) were only INR 8,742 and INR 10,377, respectively, for Russell’s viper patients. These data suggest that hospital and pharmacy costs are the most significant factors contributing to the overall treatment costs for snakebite patients. The average percentages of different treatment costs compared to the total costs for bites from diverse snake species are shown in **Table 1**.

**Table 1:** Average treatment costs of different categories of total treatment costs for each snake. All the values are shown in percentages of total treatment costs for each snake.

	Average costs of different categories of total treatment costs (%)			
Snake	Hospital	Pharmacy	Laboratory	Investigation
Common krait	33.50	53.50	10	3
Cobra	48.5	36	10	5.4
Saw-scaled viper	34	48	10.8	6.2
Russell’s viper	45.5	37.3	9.3	7.8
Non-venomous	50.9	26	13.2	9.9
Unknown	50.7	33.4	9.7	6

### Age of patients and snake species act as significant predictors of treatment costs

**Fig 3:**
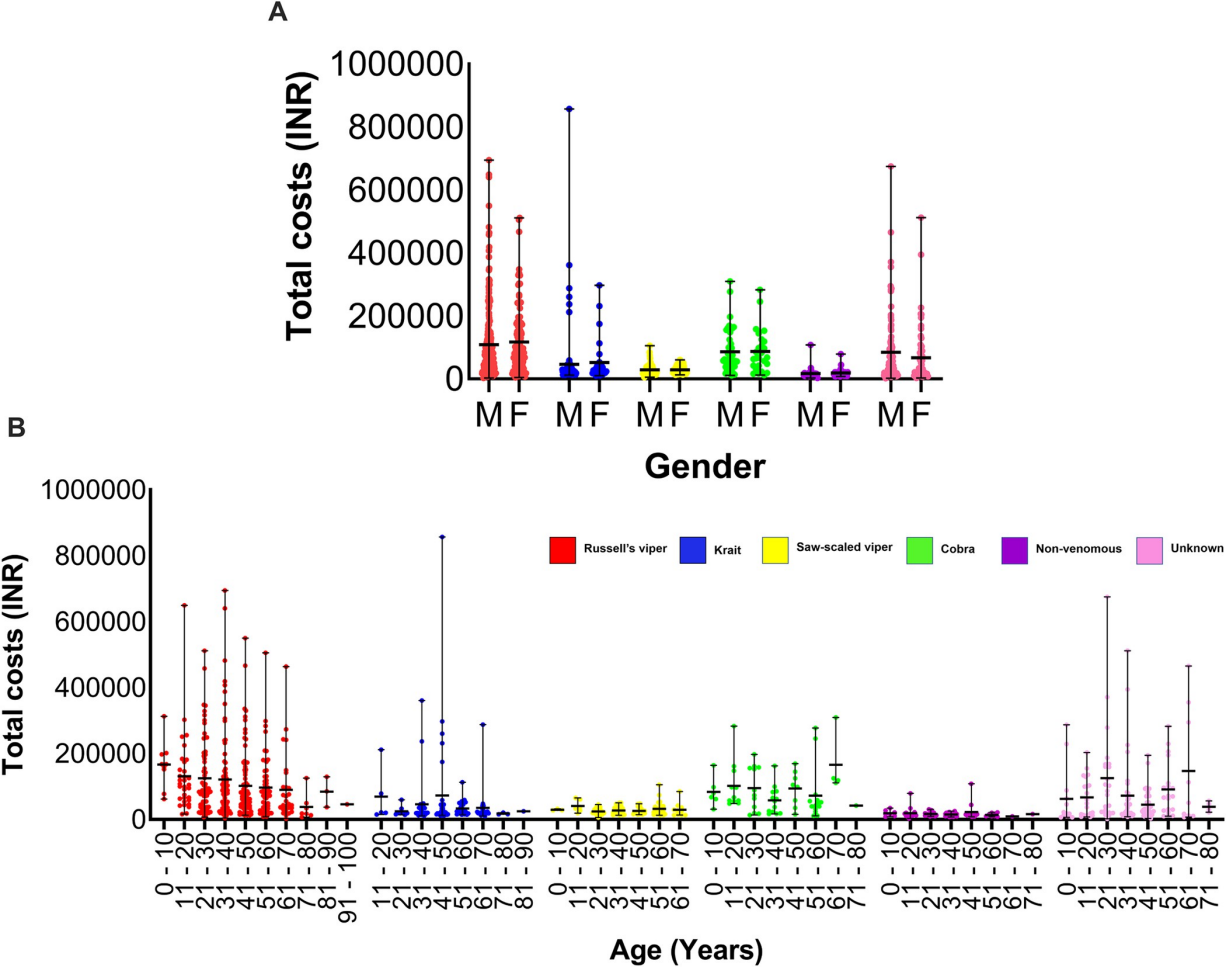
The relationship between the gender and age of snakebite patients and their total treatment costs. (**A**) The relationship between the gender of the patient and their total treatment costs for different snake species. (**B**) The patient’s age and the total treatment costs for different snakebites.

When the total treatment costs for males and females were compared, there was no significant difference between the cohorts, indicating that gender is not a significant predictor for total treatment cost [X^2^ = 0.07; df = 1; p = 0.79] (**Fig 3A**). However, when the total treatment costs of different age groups were compared, age was determined to be a significant predictor for the total treatment cost [X^2^ = 6.54; df = 1; p = 0.01] (**Fig 3B**). Similarly, when the treatment costs of patients from different snake species were compared, the offending snake was confirmed to be a significant predictor of total treatment cost [X^2^ = 332.9; df = 5; p<0.0001]. The results indicate that all snake species’ total treatment costs vary significantly from each other. However, the total treatment costs are not significantly different between Russell’s viper and the cobra, or between the unknown category and cobra. The data also indicate that the overall treatment cost decreases with age in both males and females (**Fig 3B**).

### Impact of specialist treatments on total treatment costs

All associated treatment costs for Russell’s viper bites are higher than the bites from other snakes, which can be attributed to the specialist treatments that these patients received (**Table 2**). For example, 13 patients who were bitten by Russell’s viper received dialysis and therefore, their average total treatment costs were INR 172,018 which is higher than the average (INR 111,496) total costs for Russell’s viper patients. A single patient who developed parotitis along with excessive levels of bilirubin paid a total treatment cost of INR 292,502. A total of 26 patients bitten by Russell’s viper stayed in intensive care units and they paid a total of INR 188,950. 55 Russell’s viper patients received blood transfusions and paid a total average cost of INR 139,541. 38 Russell’s viper patients had some form of operating intervention such as wound debridement and paid an average cost of INR 133,148. Similarly, seven cobra patients had wound debridement and they paid INR 91,002 which is higher than the total average (INR 86,833) costs for treating cobra patients (**Table 2**). Moreover, two patients bitten by saw-scaled vipers developed anaphylaxis and they paid higher (INR 32,457) than the average (INR 28,863) total treatment costs. Nine saw-scaled viper patients who stayed in intensive care units paid a total of INR 40,736 which is higher than the total average costs (**Table 2**). 12 patients of saw-scaled vipers had blood transfusions, and they paid INR 39,959, which is also higher than the average total treatment costs. 17 patients bitten by unknown snakes stayed in the intensive care units and paid an average of INR 83,766 which is more than the average (INR 77,342) total treatment costs. Similarly, two patients of unknown snake categories had a tracheostomy and therefore their total treatment costs were increased to INR 87,120, higher than the total average costs.

**Table 2:** Some of the specialist treatments given to patients who were bitten by different snakes and their total treatment costs. The values shown are average total treatment costs presented in INR, and the values in brackets represent the number of patients who received those treatments. ICU-intensive care unit, PCV-packed red cell volume and FFP-fresh frozen plasma.

	Average total treatment cost (INR)					
Specialist treatment	Russell’s viper	Common krait	Cobra	Saw-scaled viper	Non-venomous	Unknown
For Allergy	59,644 *(5)*	24,299 *(4)*	79,430 *(2)*	32,457 *(2)*	20,180 *(3)*	50,059 *(4)*
Dialysis	172,018 *(13)*	20,432 *(2)*	39,524 *(1)*	22,908 *(3)*	17,435 *(4)*	138,829 *(4)*
For Parotitis	292,502 *(1)*	**-**	**-**	**-**	21,254 *(1)*	**-**
For Jaundice	292,502 *(1)*	**-**	**-**	**-**	**-**	**-**
ICU	188,950 *(26)*	212,056 *(7)*	68,019 *(6)*	40,736 *(9)*	13,218 *(7)*	83,776 *(17)*
Blood transfusion	139,541 *(55)*	112,307 *(18)*	83,215 *(13)*	39,959 *(12)*	16,504 *(16)*	74,104 *(26)*
Surgeries, e.g., wound debridement	133,148 *(38)*	23,373 *(14)*	91,002 *(7)*	28,334 *(13)*	18,115 *(6)*	62,633 *(18)*
Tracheostomy	-	**-**	-	17,586 *(2)*	**-**	87,120 *(2)*
PCV	160,644 (*2)*	**-**	37,404 *(1)*	16,286 *(1)*	**-**	261,989 *(2)*
FFP	-	12,988 *(1)*	**-**	16,286 *(1)*	11,895 *(1)*	261,989 *(2)*

### The quantity of antivenom vials used is not a key factor for the total treatment cost

**Fig 4:**
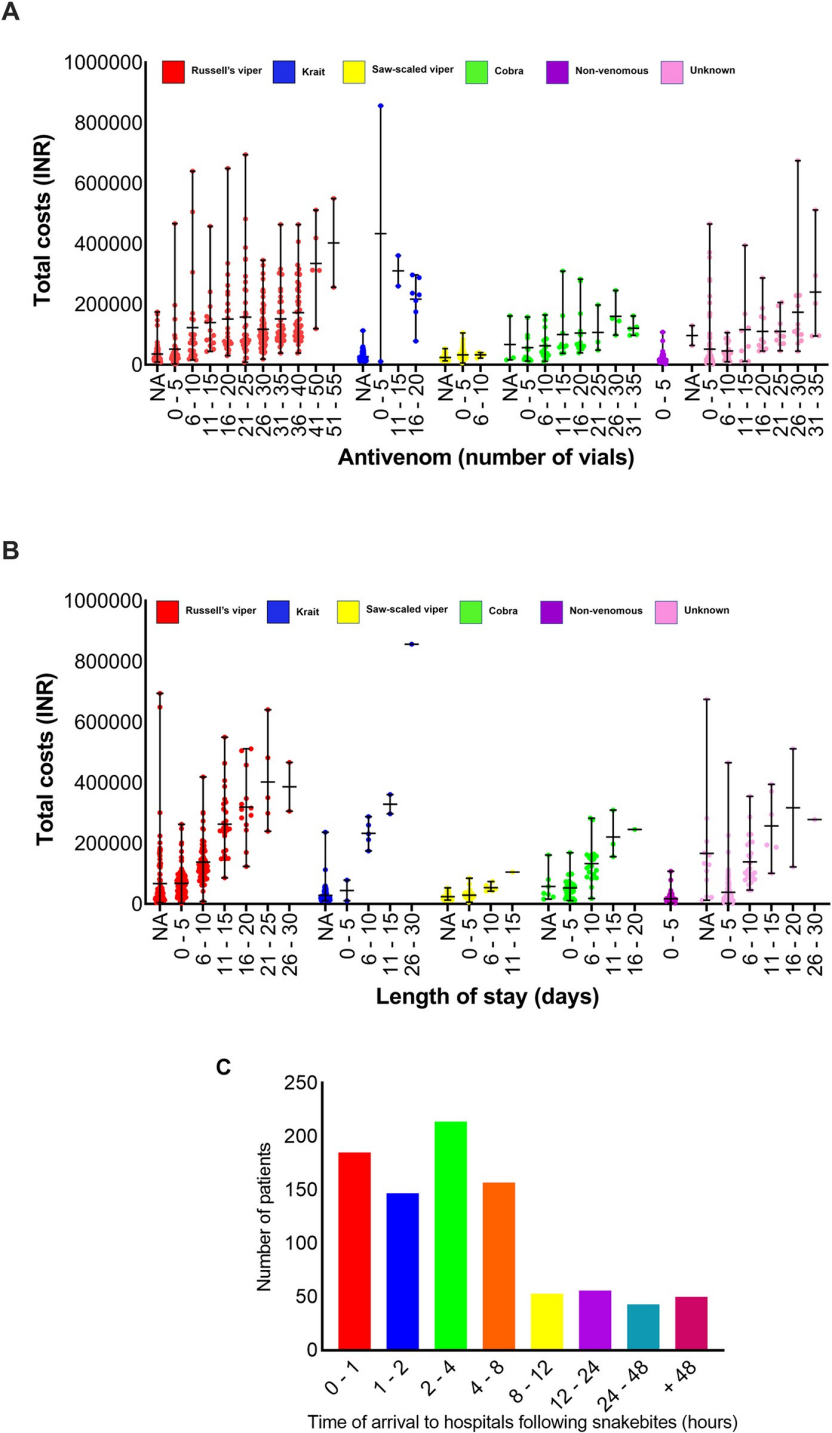
Impact of the number of vials of antivenom used, and the length of hospital stay in snakebite treatment costs. (**A**) The relationship between the number of antivenom vials received by snakebite patients and their corresponding total treatment costs for the species in question. (**B**) The length of hospital stay in days for snakebite patients and the corresponding total treatment costs. NA—‘not available’ indicates that for a small number of patients, accurate details are not available. (**C**) The number of patients arrived at the hospital at different time points following the snakebites.

We then analysed the data to determine if the volume of antivenom administered is a key predictor of total treatment costs for SBE. The number of vials of antivenom used for Russell’s viper patients varied from 3 to 50 vials (average 25; median 30). Cobra bites required 5 to 35 vials (average 16; median 15), common krait bites used 5 to 20 vials (average 16; median 20), saw-scaled viper bites used 2 to 10 vials (average 5; median 5), non-venomous snakebites used 3 to 5 vials (average 4.5; median 5) and the unknown category required 3 to 35 vials (average 17; median 15) (**Fig 4A**). The number of antivenom vials received by patients varies significantly depending on the offending snake (X^2^ = 382.485, df = 5, p = <0.0001). Neither the age (X^2^ = 0.00, df = 9, p = 0.9407) nor the gender (X^2^ = 1.484, df = 1, p = 0.223) of the patients had any significant bearing on the number of vials received. However, males received 11% (or 1.1 times) more vials than females although this was not found to be statistically significant due to large variations within the data set. The total number of antivenom vials used was calculated as a proportion of the total treatment cost, and it was found to be a lesser contributor to the total treatment cost. In most hospitals, antivenom was provided for around INR 700/vial. Therefore, its contribution to the total treatment cost ranged from INR 1,400 to INR 35,000. Compared to the total treatment costs, this is only a small factor in determining the final treatment costs in tertiary healthcare settings. The cost of an average number of antivenom vials received by Russell’s viper bite patients represents 15.7% (INR 17,500) of the total treatment cost. Similarly, it accounts for 13.1%, (INR 11,410) of cobra, 24.3% (INR 11,480) of common krait, 12.1% (INR 3,500) of saw-scaled viper, 17.9% (INR 3,150) of non-venomous and 15.7% (INR 12,110) of unknown category treatment costs. Notably, if patients received up to 10 vials of antivenom in the primary or local healthcare settings prior to their admission in tertiary care settings, then a significant decrease in the number of vials received at the tertiary private hospitals was observed (X^2^ = 10.95, df = 9, p = 0.0024).

### Impact of hospital stay, time of bites and time taken to reach hospitals on treatment costs

For Russell’s viper bites, the minimum stay in the hospital was 30 minutes and the maximum stay was 30 days (average 6.8 days; median 5) (**Fig 4B**). Similarly, patients bitten by cobras required a stay of 0.5 to 18 days (average 5.3; median 5). Common krait bite patients required between 1 to 26 days (average 9.7; median 9), non-venomous bites ranged from 2 hours to 4 days (average 1.4; median 1), and unknown bite patients ranged from one hour to 27 days (average 4; median 3). Although the length of stay in hospitals can contribute to increased treatment costs, this alone is not the sole predictor of total treatment costs for any specific snake. For example, the patients bitten by common kraits stayed an average of 9 days which is higher than any other snakebite patients but paid comparatively lower treatment costs. On the other hand, Russell’s viper bite patients who had shorter stays in the hospital paid more treatment costs than patients bitten by common kraits. Therefore, there are other contributory factors that determine the overall treatment costs for snakebite patients.

Out of the 913 patients, 252 went directly to tertiary care hospitals from where the data were collected in this study. 661 patients visited at least one local primary or secondary healthcare setting prior to reaching these tertiary care hospitals. Moreover, 185 patients arrived at the hospitals within one hour following the bite, 147 arrived between 1 and 2 hours, and 214 arrived between 2 to 4 hours following the bites (**Table 3**). Therefore, a total of 546 (60%) patients arrived at hospitals within the recommended 4-hour period following bites [15]. In addition, 157 (17%) arrived at hospitals between 4 and 8 hours after bites. The remaining patients arrived at various time points (**Fig 4C**). These data suggest that a majority of SBE patients are seeking treatment at hospitals within the recommended time either in local primary/secondary or tertiary healthcare settings.

**Table 3:** Total average treatment costs for snakebite patients who arrived at hospitals at different times following bites. The total treatment costs are shown in INR, and the number of patients in each category was shown within the brackets.

	Average total treatment cost (INR)					
Time to Hospital (hours)	Russell’s viper	Common krait	Cobra	Saw-scaled viper	Non-venomous	Unknown
0–1	102,277 *(56)*	39,779 *(38)*	82,898 *(15)*	25,615 *(24)*	18,675 *(20)*	80,652 *(32)*
1–2	120,366 *(58)*	35,199 *(22)*	69,623 *(13)*	30,245 *(21)*	14,600 *(15)*	53,955 *(18)*
2–4	110,624 *(87)*	48,095 *(31)*	97,119 *(16)*	28,106 *(23)*	19,783 *(18)*	80,368 *(39)*
4–8	115,202 *(60)*	34,741 *(24)*	58,066 *(14)*	30,163 *(19)*	15,065 *(11)*	63,718 *(29)*
8–12	85,032 *(21)*	66,690 *(9)*	113,061 *(4)*	31,417 *(4)*	21,793 *(7)*	93,014 *(8)*
12–24	126,879 *(22)*	30,425 *(6)*	136,822 *(4)*	31,325 *(9)*	16,569 *(4)*	132,935 *(11)*
24–48	124,590 *(20)*	21,469 *(1)*	277,473 *(1)*	28,460 *(1)*	18,782 *(8)*	60,110 *(12)*
+ 48	101,134 *(25)*	436,326 *(2)*	37,404 *(1)*	28,863 *(6)*	10,946 *(4)*	82,501 *(12)*

We found that the time of arrival is not a significant predictor of total treatment costs for most SBE patients (**Table 3**). However, the data indicate that Russell’s viper patients who arrived after 12 hours following bites paid more (INR 126,879) than the total average costs (INR 111,496) for Russell’s viper patients although it is not significant. Similarly, common krait patients who arrived eight hours following bites paid more (INR 66,690) than the total average (INR 47,276) treatment costs. Cobra patients who arrived after 4 hours following bites paid more (INR 124,942) than the average total costs (INR 86,833). Saw-scaled viper patients who arrived four hours following bites paid more (INR 30,968) than the average total treatment costs (INR 28,863). Notably, there was a significant difference (p<0.0001; df = 7; f = 7.155) when comparing the total treatment costs for common krait patients who arrived after 48 hours following bites (INR 436,326) with patients who arrived within one hour (INR 39,779) following bites although there were only two patients admitted to the hospitals after 48 hours. Similarly, there was a significant (p = 0.0291; df = 7; f = 2.343) difference in total treatment costs of cobra patients who arrived after 24 hours (INR 277,473) compared to patients who arrived within one hour (INR 82,898) although only one cobra patient arrived after 24 hours. These data reemphasise that the delay in seeking hospital treatment is a key factor in contributing to increased treatment costs.

Moreover, when analysing the total treatment costs of patients who were bitten during day times (between 6 am and 6 pm), early evenings (6 pm to 10 pm) and late night (10 pm to 6 am), there was no significant difference in the total treatment costs for Russell’s viper, saw-scaled viper, and cobra patients although the values are high for cobra and saw-scaled viper patients who arrived after 10 pm (**Table 4**). However, common krait patients who were bitten after 10 pm paid significantly more (INR 138,575) than the total average treatment costs (INR 47,276). The increase in treatment costs in late nights could be due to high transport costs, and investigation charges as the specialists may need to come from home or other hospitals for expert investigation and emergency intubation. As the common krait is a nocturnal snake, its bites are likely to be high at night times leading to late-night hospital admissions.

**Table 4:** Total average treatment costs for snakebite patients who were bitten at different times of the day/night. The treatment costs are shown in INR and the number of patients is shown in brackets.

	Average total treatment cost (INR)					
Time of bite	Russell’s viper	Common krait	Cobra	Saw-scaled viper	Non-venomous	Unknown
06:00–10:00	98,601 *(67)*	20,342 *(14)*	82,233 *(14)*	29,400 *(17)*	14,649 *(15)*	62,869 *(24)*
10:00–14:00	115,039 *(70)*	32,840 *(27)*	71,657 *(18)*	25,471 *(23)*	17,749 *(18)*	103,124 *(31)*
14:00–18:00	132,837 *(69)*	31,865 *(34)*	97,357 *(13)*	28,567 *(26)*	16,439 *(19)*	72,987 *(29)*
18:00–22:00	112,646 *(98)*	40,165 *(38)*	87,910 *(16)*	29,468 *(24)*	20,787 *(29)*	66,320 *(56)*
22:00–02:00	107,039 *(20)*	168,272 *(7)*	117,142 *(6)*	34,046 *(9)*	10,206 *(5)*	87,585 *(9)*
02:00–06:00	86,575 *(17)*	108,878 *(12)*	13,655 *(1)*	27,891 *(6)*	21,767 *(1)*	55,543 *(6)*

### The hospital care for nearly 80% of SBE patients cost INR 100,000 or less for their treatments

**Fig 5:**
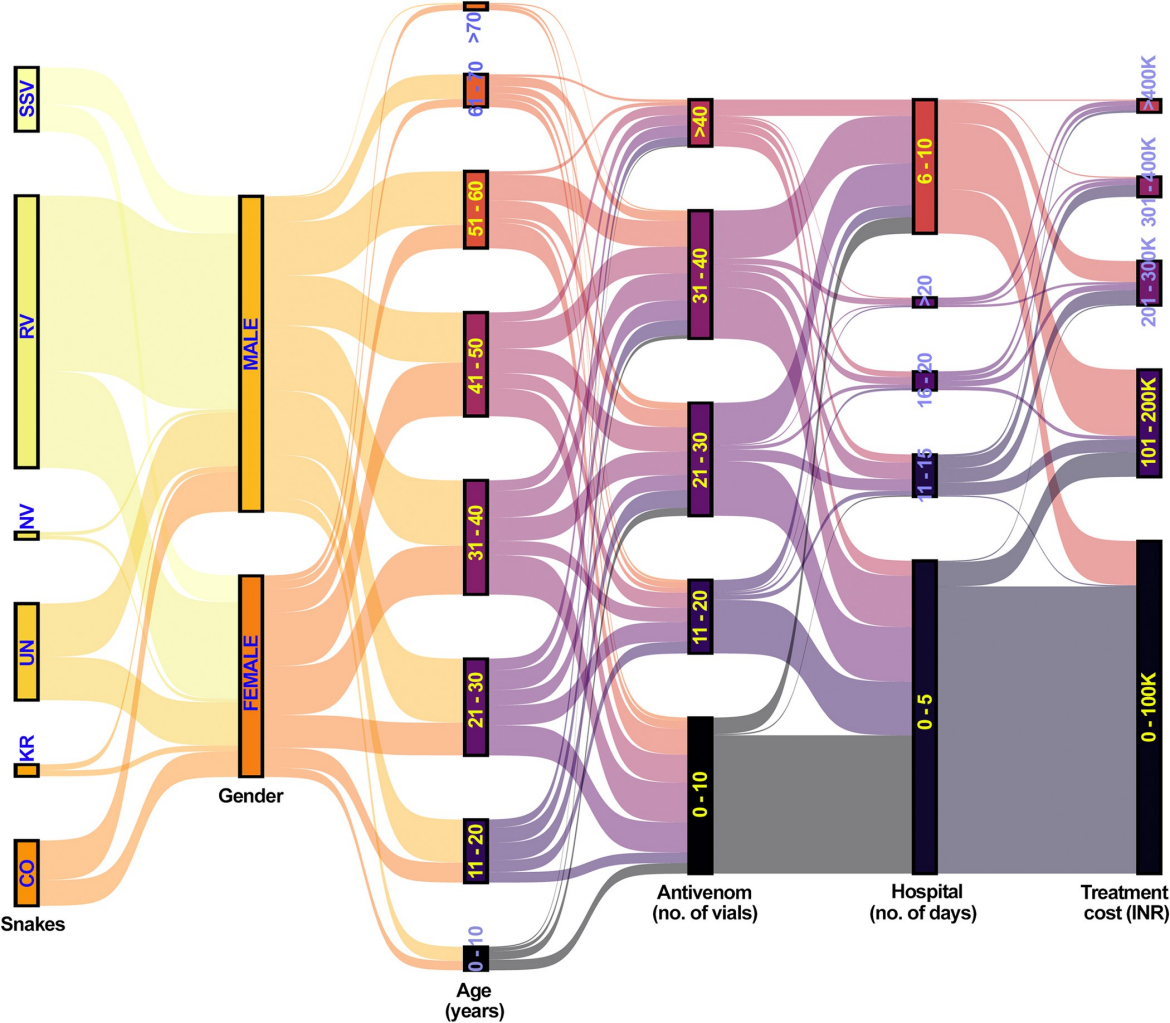
A Sankey plot showing the relationships between treatment costs and various parameters analysed in this study. RV—Russell’s viper, CO—cobra, KR—common krait, UN—unknown, NV—non-venomous snakes and SSV—saw-scaled viper.

Finally, the total amount of treatment costs was analysed for all snakebite patients. This reveals that 711 (78%) patients paid INR 100,000 (~GBP 1000 or USD 1200) or less for their hospital treatment. This includes 223 (63%) Russell’s viper, 49 (71%) cobra, 123 (93%) common krait, 107 (99%) saw-scaled viper, 86 (99%) non-venomous and 123 (76%) unknown bite patients. 120 (13%) patients paid between INR 100,001 to INR 200,000 for their treatment. 47 (5%) patients paid between INR 200,001 to INR 300,000, and 19 (2%) paid INR 300,001 to INR 400,000 for their treatments. The higher cost of more than INR 400,000 was paid by a minority of patients. These data suggest that despite gender, age, the offending snake species, time of the bite, time taken to reach tertiary care hospitals, administration of antivenom and specialist treatments used, around 80% of patients can be treated with INR 100,000 or less in private tertiary healthcare settings (**Fig 5**).

## Discussion

SBE affects rural communities in several countries specifically in South Asia, Africa and Central and South America [2, 3]. The World Health Organisation (WHO) has developed a strategic road map comprising four key pillars to reduce SBE-induced deaths and disabilities by half in 2030. To achieve this ambitious target, we need to initiate trans-disciplinary research on various aspects associated with SBE to establish its true impact on vulnerable communities. Notably, strengthening the health system is one of the key pillars of the WHO’s SBE strategies. Therefore, it is critical to understand the wider context of healthcare systems, their operations, training needs and the costs of treating SBE in vulnerable countries [31]. Healthcare costs are a major issue in many parts of the world specifically in low- and middle-income countries [32]. While some countries provide healthcare free of charge for SBE patients, in other places people pay for their treatment out of pocket. Among poor communities living in rural areas of developing countries, the affordability of healthcare is a major issue [32]. Since SBE is an unexpected accident as well as an occupational health hazard, the treatment costs can cause significant socioeconomic impacts on patients and their families for generations [10,12,33]. As shown in our previous studies [10,18], SBE treatment costs vary widely based on the nature of the bite and the type of treatments involved. In most cases, the patients do not have any health insurance to cover their treatment costs [10]. This results in patients taking out loans for treatment costs from private lenders as the nationalised banks often do not offer such loans to cover medical expenses. The subsequent economic hardships force some patients to sell their homes, valuables, and other assets to cover costs [10]. In some cases, they are forced to withdraw their children from education to start working to bring in more money to cover household costs and alleviate the financial burden that SBE has caused. Hence, SBE treatment costs act as a key factor in instigating socioeconomic impacts on patients and their families, inducing a cycle of poverty [10,11]. However, the availability of data on a range of treatments involved and their costs for SBE in many parts of the world specifically in India is highly limited [34]. Therefore, this study analysed the treatment costs in a broad sample of private tertiary healthcare settings in Tamil Nadu, India to establish the key factors associated with SBE treatment costs that will aid in developing strategies to minimise these costs and enable SBE patients to seek prompt treatment.

India suffers around 58,000 annual deaths due to SBE even though the medical facilities are advanced in urban areas [8]. Healthcare in India is provided by both government and private parties including some charitable organisations [12]. In India, the state and central governments have an excellent architecture in their healthcare system including village health nurses, primary health centres, taluk hospitals, district general hospitals and medical college hospitals. These healthcare facilities have the required facilities and expertise to provide free healthcare for everyone [12]. For a range of different reasons, a vast majority of people seek treatment in private healthcare settings even for mild ailments. Similar to the government’s healthcare system, private parties run small primary care clinics in rural areas and secondary (with limited facilities), tertiary (hosts all necessary facilities, equipment and expertise) and super speciality (well-advanced facilities, equipment and expertise) hospitals in urban areas [12, 35]. In addition, local pharmacies are mostly managed by private individuals, and they often treat mild ailments with over-the-counter medications. People must pay for their treatments and medicines in all private healthcare settings. Based on the type of illness, nature of treatments and the provider used, the treatment costs vary widely [35]. Due to the broad range of complications arising from snake venoms, SBE patients are mostly treated in tertiary healthcare settings to tackle the issues promptly.

In this study, the data collected from participating private tertiary care hospitals were divided into several categories (hospital, pharmacy, investigation, and laboratory costs) to underpin the key contributors to the overall treatment costs. This has simplified the discussion about the nature of treatments involved and the rationale for expensive treatments. The hospital costs that cover intensive/critical care units, wards, operating theatres, and surgical procedures appeared to be the major contributor to total treatment costs. Pharmacy cost as a category covered all medications used during the treatment of SBE patients including antivenom and was the second significant contributor to SBE treatment costs. Each vial of antivenom only costs around INR 700 to INR 1,000 but additional costs such as antibiotics, blood products, consumables and other relevant materials are key contributors to treatment costs. Since the government sets the price for antivenom in India, this helps to minimise the costs of antivenom to the patients. The laboratory investigation and the fees for clinicians including specialists were found to be the least significant contributors to the total treatment costs compared to hospital and pharmacy costs. In this study, nearly 95% of people did not have medical insurance and therefore, they had to pay the full treatment costs. However, a small number of people had medical insurance although it did not cover the full treatment costs, so they still had to contribute towards the total treatment costs. In some hospitals, small discretionary discounts were offered based on the financial situation of patients, however, this is not the most common option offered to all patients.

Among the different snakebites analysed in this study, Russell’s viper bites appear to incur greater treatment costs compared to the other snakes analysed. The amount of venom injected and therefore, resulting complications following Russell’s viper bites vary widely. In some patients, a small amount of tissue damage and coagulation abnormalities occur, however, in others, it results in extensive tissue damage which demands specialist interventions. Surgical procedures such as debridement, fasciotomy and amputation require operating theatres and consumables, which results in increased costs. For example, a simple debridement and fasciotomy may cost around INR 5000 to cover staff and operating theatre costs. However, amputation of a limb may increase this cost up to ten times (INR 50,000). Due to the impacts of Russell’s viper venom on the coagulation system, the patients often need plasma or whole blood transfusions, and other related products [36]. Notably, the unknown category shown in this study is likely to include many bites from Russell’s vipers as these are most frequently encountered. The bites from cobra and common krait often may need mechanical ventilation support for days to weeks [37]. While cobra patients may need ventilation only for a few days, common krait bite patients may need up to a week or longer [14,20]. The clinicians believe that at least 1 in 10 SBE patients needs ventilation support as part of their treatment. The ventilation charges vary widely from around INR 3500 to INR 20,000 every day based on the type of hospital and the time. This ultimately increases the total treatment costs for SBE patients. For example, a patient who arrives at a hospital promptly (within 30 minutes) after the bite with only prolonged coagulopathy without any other complications may cost around INR 20,000 to INR 25,000 to treat. However, a patient with local swelling, fasciotomy and 20 vials of antivenom is likely to cost around INR 35,000. Acute kidney injury necessitates dialysis which costs around INR 2000 to INR 5,000 (based on the hospital) per cycle, with patients often requiring 3 to 15 cycles over two to three weeks. Hospitals normally charge around INR 2000 to INR 10,000 per day for intensive care units, and the SBE (specifically Russell’s viper) patients may need care in this unit for 3 to 8 days. Similarly, for wards, hospitals charge anything between INR 500 to INR 3000 based on the type of ward the patients choose. Generally, treating saw-scaled viper bites in Tamil Nadu costs less as it often requires less than the minimum 10 vials of antivenom for SBE in the rest of India and pain management for these bites is effective. The treatment for non-venomous snakebites costs around INR 1000 to INR 2000 as they only necessitate monitoring of the patients for up to 24 hours and some basic measurement of blood parameters along with infection management when needed. The data from this study suggest that for any venomous snakebites, the delay in seeking treatment may exacerbate complications and further increase treatment costs. Therefore, SBE patients should seek immediate medical care after the bite to minimise the threat to life, complications, and subsequent treatment costs. This has also been reported in a previous study where longer hospital stays resulted in higher treatment costs [38].

Snakebite patients and their families and communities have different perspectives in seeking prompt hospital treatment. In some places, snakebite has been considered a fate, and therefore, they should not seek any treatment. For several individuals, the traditional treatment is the only solution for SBE although this significantly delays the hospital treatment [16,39,40]. Similarly, there are several other misconceptions about snakes and SBE treatments [41]. Notably, several people are intimidated to seek prompt medical care due to high treatment costs in private hospitals [10]. Therefore, more healthcare policies should be developed to tackle various issues associated with SBE treatment costs. For example, intense public awareness activities are required to educate people about the identification of locally available medically important venomous snakes and encourage patients to seek prompt care as arriving at the hospitals earlier will reduce the treatment costs [12,15,27,40,42]. The delay in seeking treatment is not only exacerbating envenomation effects and treatment costs, but it is also significantly increasing the long-term health consequences, which may ultimately result in further socioeconomic impacts [43]. Notably, the policies to provide free medical care in private healthcare settings or health insurance to fully cover the treatment for SBE will significantly encourage people to arrive at hospitals promptly after the bite. It is often inevitable to arrive at late night due to the nature of SBE (e.g., common krait bites at night times), and therefore, clinicians and hospitals should consider charging the same amount even for patients who are arriving at late night as SBE mainly affects poor rural communities. The use of intensive care units is also critical and indeed, some patients can be managed with only supportive measures such as ventilation support in such units even in the absence of antivenoms [23]. Therefore, the hospitals should consider reducing the charges for intensive care units for SBE. People prefer immediate access to emergency services, which are often difficult to reach late at night. Notably, there are numerous remote and tribal villages not just in India but also in other countries such as Brazil [44,45] and Kenya [39] without proper road facilities where people have to carry the SBE patients (as well as others) to the nearest facilities. Although there may not be an immediate solution for this issue, rural healthcare workers should be trained to empower them to provide appropriate first aid and administer a few vials of antivenom in the closest setting that is possible, before transporting the patients to distant hospitals [39,46,47]. Training for rural healthcare professionals is also critical for the early assessment of patients, ascertaining envenoming compared to the dry and non-venomous bites [48] and their timely referral to tertiary care settings [27,49]. Early intervention using appropriate adjunct therapy may also significantly reduce the envenomation effects and subsequent treatment costs [34]. Governments, non-governmental organisations and private hospitals could offer SBE first aid kits containing some key materials such as pressure bandages, gauze cloths, and painkillers to support the patients in such remote locations [46]. This study has noted that pharmacy costs are high, and this can be minimised if some leading pharmaceutical companies offer key medications such as antivenoms, antibiotics and blood products at a reduced cost exclusively for SBE patients. It is also important to ensure adequate antivenom production and efficient supply including in rural healthcare settings to allow easy access [17,50–52].

Due to the complex nature of this study, it has several limitations. Since the treatments in government hospitals are provided free of cost to patients, we did not collect the actual costs incurred by the governments to treat SBE in these settings as this was outside of the scope of this work. However, it would have been helpful to analyse these data to determine the contributions of governments to tackle SBE. This study was conducted by collecting only direct treatment costs from 913 snakebite patients who were treated in 10 different private tertiary care hospitals within Tamil Nadu. Therefore, future studies with more patients and hospitals including the ones managed by the governments and charitable organisations are required to get a better understanding of the treatment costs for SBE across Tamil Nadu. Similar studies are also warranted in other states of India as the healthcare system may vary widely across the country. Moreover, we mainly analysed the data under four major categories due to the small sample size specifically for snakes other than Russell’s viper. A larger sample size would allow further characterisation of the treatment costs, allowing for a more in-depth analysis to be performed on the specific factors associated with increased treatment costs. We also did not thoroughly analyse the number of antivenom vials administered in local healthcare centres prior to arriving at these tertiary care hospitals due to the poor memory of patients or the unavailability of these data in several cases. These data will add more to estimate the overall treatment costs for SBE. Therefore, data and costs presented in this study should only be considered as guidance for further studies and policy development as this may vary widely in different hospitals in India and other countries. Moreover, inflation, increasing costs of medications and equipment and availability of medications may ultimately play a role in the increased treatment costs for SBE. The indirect costs associated with SBE treatments were not analysed in this study, and this is another key factor that should be considered in future studies. This study did not discriminate between brands of antivenom used as national standards dictate the potency requirements across brands. Additionally, we did not attempt to analyse the social patterns associated with gender, age, or occupational differences in the presentation of different kinds of snakebites although these are areas of interest for epidemiological and educational purposes. The use of clinical symptoms by treating clinicians was mainly used to ascertain the snake species, and this could have been inaccurate in some cases. Notably, women may not have visited hospitals in a similar manner to men although there is no evidence to ascertain this notion. However, this is another key factor that should be addressed in future.

## Conclusions

Overall, this study suggests that there are numerous factors associated with the treatment costs for SBE in private tertiary care hospitals. However, it appears that a vast majority (~80%) of SBE patients can be treated with around INR 100,000 or less. Further increases in costs are attributable to complications resulting in operative interventions, dialysis, prolonged ventilation support, extended hospitalisation, and medications. Major healthcare insurance providers could consider SBE as a disease of poverty and offer cheap insurance schemes to cover these people from unexpected SBE treatment costs. Members of the public should also consider taking out these insurance schemes by paying a small fee, protecting themselves from SBE treatment costs. Necessary awareness should be created for members of the public to emphasise the need for taking such medical insurance to access prompt care. Moreover, there are currently no strict regulations or guidelines for SBE treatment costs in private healthcare settings. This results in hospitals deciding on the treatment cost themselves and can often appear unjust to the patients. While patients struggle to survive SBE complications, the treatment costs often come as another major shock, leading to serious downstream socioeconomic impacts. The societal impacts arising from SBE-associated financial burden can be a contributor to causing a shift in the social status of patients and their families within their communities for generations to come. Therefore, the health authorities could investigate this system and try to balance it. The prime minister’s and chief minister’s comprehensive insurance schemes in India could also be extended to more people and cover the entire treatment costs associated with SBE as it mostly affects poor rural farmers. India is a nation that largely relies on its agricultural and related workforce, and therefore, protecting these key workers from unexpected medical costs such as due to SBE is a priority. A key finding of the study is consistent with other studies in that antivenom is a significantly minor driver of costs while still being the only pharmacological preparation known to be effective in the treatment of snakebites [34,53]. The outcomes of this study may be useful to most if not all healthcare systems worldwide to improve the clinical management and accessible treatment for SBE. However, there may be several region- and country-specific issues that need to be considered while adapting these data for different regions, but these data will act as a basis for further discussions. In addition, this data will form the basis to support the policy recommendations within the countries and by the WHO to facilitate accessible treatments for SBE. Some of the key policy recommendations would be to provide health insurance and key medications free of cost or at a cheaper rate for all vulnerable communities. Together, these policies may aid in achieving the goals of the WHO to reduce SBE-induced deaths and disabilities by 50% in 2030 [34,53,54].
